# NEO: NEuro-Inspired Optimization—A Fractional Time Series Approach

**DOI:** 10.3389/fphys.2021.724044

**Published:** 2021-09-21

**Authors:** Sarthak Chatterjee, Subhro Das, Sérgio Pequito

**Affiliations:** ^1^Department of Electrical, Computer, and Systems Engineering, Rensselaer Polytechnic Institute, Troy, NY, United States; ^2^MIT-IBM Watson AI Lab, IBM Research, Cambridge, MA, United States; ^3^Delft Center for Systems and Control, Delft University of Technology, Delft, Netherlands

**Keywords:** optimization, time series processes, iterative optimization algorithms, long memory time series, fractional calculus

## Abstract

Solving optimization problems is a recurrent theme across different fields, including large-scale machine learning systems and deep learning. Often in practical applications, we encounter objective functions where the Hessian is ill-conditioned, which precludes us from using optimization algorithms utilizing second-order information. In this paper, we propose to use fractional time series analysis methods that have successfully been used to model neurophysiological processes in order to circumvent this issue. In particular, the long memory property of fractional time series exhibiting non-exponential power-law decay of trajectories seems to model behavior associated with the local curvature of the objective function at a given point. Specifically, we propose a NEuro-inspired Optimization (NEO) method that leverages this behavior, which contrasts with the short memory characteristics of currently used methods (e.g., gradient descent and heavy-ball). We provide evidence of the efficacy of the proposed method on a wide variety of settings implicitly found in practice.

## 1. Introduction

Many problems in today's world can be modeled as optimization problems where we seek to find the solution to an unconstrained optimization problem with an objective function *f* :ℝ^*n*^ → ℝ (Bishop, 2006; Sra et al., 2012), which is a real-valued function of *n* real variables. For instance, in a learning problem, we aim to minimize a loss index that measures the performance of a neural network (NN) on a data set. Often, the loss index is composed of an error term and a regularization term that evaluates how well the NN fits the data set and discourages overfitting, respectively (Scholkopf and Smola, 2001). Besides controlling overfitting, the regularization term can also be designed to control the complexity of a NN, e.g., by reducing the number of non-zero weights *w* ∈ ℝ^*n*^ (LeCun and Bengio, 1995).

Notwithstanding the above, most optimization problems do not possess numerically viable closed-form solutions (Boyd and Vandenberghe, 2004; Nocedal and Wright, 2006). Furthermore, due to the ever-increasing dimensionality of data used to test a variety of optimization problems, iterative algorithms need to be employed to attain an approximate solution. At the core of the iterative algorithms, we can commonly find three key ingredients (Bertsekas, 1997; Nocedal and Wright, 2006): (*i*) a descent direction *d* ∈ ℝ^*n*^, (*ii*) a learning rate (or, step) α ∈ ℝ; and (*iii*) local spatial information across different variables (i.e., *w* ∈ ℝ^*n*^). In a nutshell, the iterative algorithms can be written as


(1) $$\begin{array}{r} w_{k+1}=w_{k}+\alpha_{k}d_{k},\;k=0,1,\ldots , \end{array}$$

where the descent direction $d_{k}\in {\mathbb{R}}^{n}$ and the step α_*k*_ ∈ ℝ might change over time step *k*. Descent directions can be computed using one of the following options:

- Gradient descent, *d*_*k*_ = −∇*f*(*w*_*k*_), where ∇ denotes the first-order derivative (Nocedal and Wright, 2006);
- Stochastic gradient descent, $d_{k}=-\tilde{\nabla }f\left(w_{k}\right)$, where sampled points and the approximate notion of the derivative are used to determine a possible descent direction and where $\tilde{\nabla }$ represents the first-order derivative calculated from either a single point in the data set or an arbitrarily selected subset of the entire data set (an approach referred to in the literature as mini-batch, Saad, 2009);
- Newton's method, $d_{k}=-{\left(Hf\left(w_{k}\right)\right)}^{-1}\nabla f\left(w_{k}\right)$, where *Hf*(*w*_*k*_) denotes the Hessian matrix at *w*_*k*_, i.e., the second-order derivative (Boyd and Vandenberghe, 2004); and
- Quasi-Newton methods, $d_{k}=-B_{k}^{-1}\nabla f\left(w_{k}\right)$, where the *B* is an approximation of the Hessian matrix, e.g., using the Broyden-Fletcher-Goldfarb-Shanno (BFGS) method (Dennis and Schnabel, 1996).

Simply speaking, the second-order derivative captures (local) spatial properties of the function through its cross-derivatives and geometrically corresponds to a quadratic function approximation of the function that we are optimizing. Subsequently, first-order iterative methods (e.g., gradient descent and stochastic gradient descent) are slower than second-order iterative methods (e.g., Newton and quasi-Newton) but require less computational power and memory storage. Recent literature in this direction has been largely focused on the hybridization of these techniques in context-based scenarios (Schraudolph et al., 2007; Roux et al., 2012; Johnson and Zhang, 2013; Cevher et al., 2014; Defazio et al., 2014; Byrd et al., 2016; Moritz et al., 2016; Rodomanov and Kropotov, 2016; Zhang and Gu, 2016; Mokhtari et al., 2018; Paternain et al., 2019).

An alternative to speeding up the convergence while not resorting to second-order methods is to consider memory in the iterative process. Specifically, consider


(2) $$\begin{array}{r} w_{k+1}=w_{k}+\alpha_{k}d_{k}+m\left(w_{k-1},\ldots ,w_{k-T}\right),\;k=0,1,\ldots , \end{array}$$

where *m*:ℝ^*n*×*T*^ → ℝ^*n*^ is a function of the previous instances of the parameters up to *T* time steps in the past. A particular case is the so-called heavy-ball method proposed by Polyak (Polyak, 1964) that builds on momentum-based physical intuition (that actually might not converge, Lessard et al., 2016) and later made more formal by Nesterov in today's celebrated accelerated convergence methods (Nesterov, 2013). Nesterov's proof techniques abandoned physical intuition and devised the method of estimate sequences to verify the correctness of these momentum-based methods. Nonetheless, researchers have struggled to understand the foundations and scope of the estimate sequence methodology since the proof techniques are often viewed as “algebraic tricks” that are only applicable to some classes of functions.

Consequently, a more complete arsenal of tools is needed to understand the convergence of first-order methods that consider memory. To address this issue, recent literature has leveraged insights and tools available in dynamical systems theory, as, in the limit, the iterative algorithm is a dynamical system in which evolution is described by ordinary differential equations (Su et al., 2014; Hardt et al., 2016; Lessard et al., 2016; Wilson et al., 2016; Hu and Lessard, 2017; Fazlyab et al., 2018; Zhang et al., 2019). These accelerated methods are also often driven by an exogenous signal (or control input) that regulates both the asymptotic convergence to the minimum and also the convergence rate.

In this paper, we seek to leverage fractional-order calculus (Oldham and Spanier, 1974; Baleanu et al., 2011, 2012; Ortigueira, 2011) to develop a new iterative optimization algorithm. Fractional derivatives and fractional-order processes have been widely used to model phenomena having long-term memory in the context of neurophysiological data (Lundstrom et al., 2008; Xue et al., 2016). Additionally, autoregressive fractionally integrated moving average (ARFIMA) time series processes are successfully able to model and explain a wide variety of biological phenomena (Ionescu et al., 2017), particularly phenomena with long-term memory and power law dependence of trajectories (Miller et al., 2009), and have been used in contexts such as the prediction and forecasting of financial market data (Bukhari et al., 2020). We are inspired by the recent spate of successes that fractional-order based models have enjoyed in the context of neuronal data (Teka et al., 2014) as well as increasing evidence presented to explain the intricate relationships between the neural-like architectures used with great success in deep learning and their relationships with systems neuroscience (Richards et al., 2019).

Consequently, we propose a novel iterative method termed as NEO (NEuro-inspired Optimization). At each step, NEO models the local evolution (i.e., determines the ARFIMA model that best describes the local curvature) and takes the argument that attains the lowest predicted values as the next iteration point. We provide the proof of convergence of the proposed method and numerical evidence of the efficacy of NEO on a variety of problem settings implicitly found in practice. Additionally, we notice that NEO only requires the values of the objective at the points, without the need to compute derivatives, and without the explicit need to tune a step size. Furthermore, our simulations suggest that the major advantages of NEO lie in cases where the Hessian is ill-conditioned, which is particularly important in the context of several real-world problems, for instance, neural networks (Saarinen et al., 1993).

## 2. Fractionally Integrated Time Series Processes

A class of stationary long-term processes *z*_*t*_ modeled as *autoregressive fractionally integrated moving average* (ARFIMA) processes are described by


(3) $$\begin{array}{r} \phi \left(B\right){\left(1-B\right)}^{d}z_{t}=\theta \left(B\right)a_{t}, \end{array}$$

for *d* ∈ ℝ, *d* being the *fractional differencing parameter*. Here, *a*_*t*_ is a white noise sequence having zero mean and bounded variance $\sigma_{a}^{2}$, the polynomial equations


(4) $$\begin{array}{r} \phi \left(B\right)=1-\overset{p}{\underset{i=1}{\sum }}\phi_{i}B^{i}=0 \end{array}$$

and


(5) $$\begin{array}{r} \theta \left(B\right)=1+\overset{q}{\underset{i=1}{\sum }}\theta_{i}B^{i}=0 \end{array}$$

have roots that are greater than unity in absolute value, and *B* is the *backward shift operator* with the property $B^{m}z_{t}=z_{t-m}$. The general form of the processes that can be represented using (3) are called ARFIMA(*p, d, q*) processes (Box et al., 2015).

For *d* > −1, we can employ the binomial expansion formula to explicitly expand the operator (1 − *B*)^*d*^ as


(6) $$\begin{array}{r} {\left(1-B\right)}^{d}=\overset{\infty }{\underset{j=0}{\sum }}\pi_{j}B^{j}, \end{array}$$

with π_0_ = 1, and $\pi_{j}=\frac{\Gamma \left(j-d\right)}{\Gamma \left(j+1\right)\Gamma \left(-d\right)},j=1,2,\ldots ,$ with Γ(·) being the Gamma function defined by $\Gamma (x)=\int_{0}^{\infty }s^{x-1}e^{-s}\text{ d}s$ for all complex numbers *x* with ℜ(*x*) > 0. We note that the weighting coefficients π_*j*_ can be defined recursively in *j*. Further, even though the binomial expansion of the operator (1 − *B*)^*d*^ consists of an infinite number of terms, in practice we will always consider an approximation that still preserves the dependency of parameters described.

More generally, ARFIMA processes can generalize ordinary autoregressive moving average (ARMA) models in the following way. Given a time series, we can carry out the following steps to obtain the parameters in (1):

- Apply fractional differencing on the original time series and note the order of the fractional difference *d* that makes the time series (close to) wide-sense stationary;
- Determine the ARMA parameters $p,q,{\left\{\phi_{i}\right\}}_{i=1}^{p}$, and ${\left\{\theta_{i}\right\}}_{i=1}^{q}$ using the (fractionally) differentiated time series;
- Perform a forecast for a requisite number of steps ahead in time with these ARMA terms; and;
- Fractionally integrate the forecasted ARMA data to obtain the forecast of the ARFIMA process. Note that fractional integration may be interpreted as fractional differentiation but with a fractional differencing parameter of −*d*.

## 3. The NEuro-Inspired Optimization (NEO) Method

In what follows, we outline the details of the NEO iterative optimization method that seeks to determine the solution to an unconstrained optimization problem. At each step, NEO models the local evolution (i.e., determines the ARFIMA model that best describes the local curvature) and takes the argument that attains the lowest predicted values as the next iteration point.

In order to develop the intuition about the NEO method, let us consider a pedagogical example – i.e., we seek to determine the solution to the following unidimensional unconstrained optimization problem


(7) $$\begin{array}{r} x^{⋆}=\underset{x\in \mathbb{R}}{\mathrm{argmin}}x^{2}. \end{array}$$

**Figure 1:**
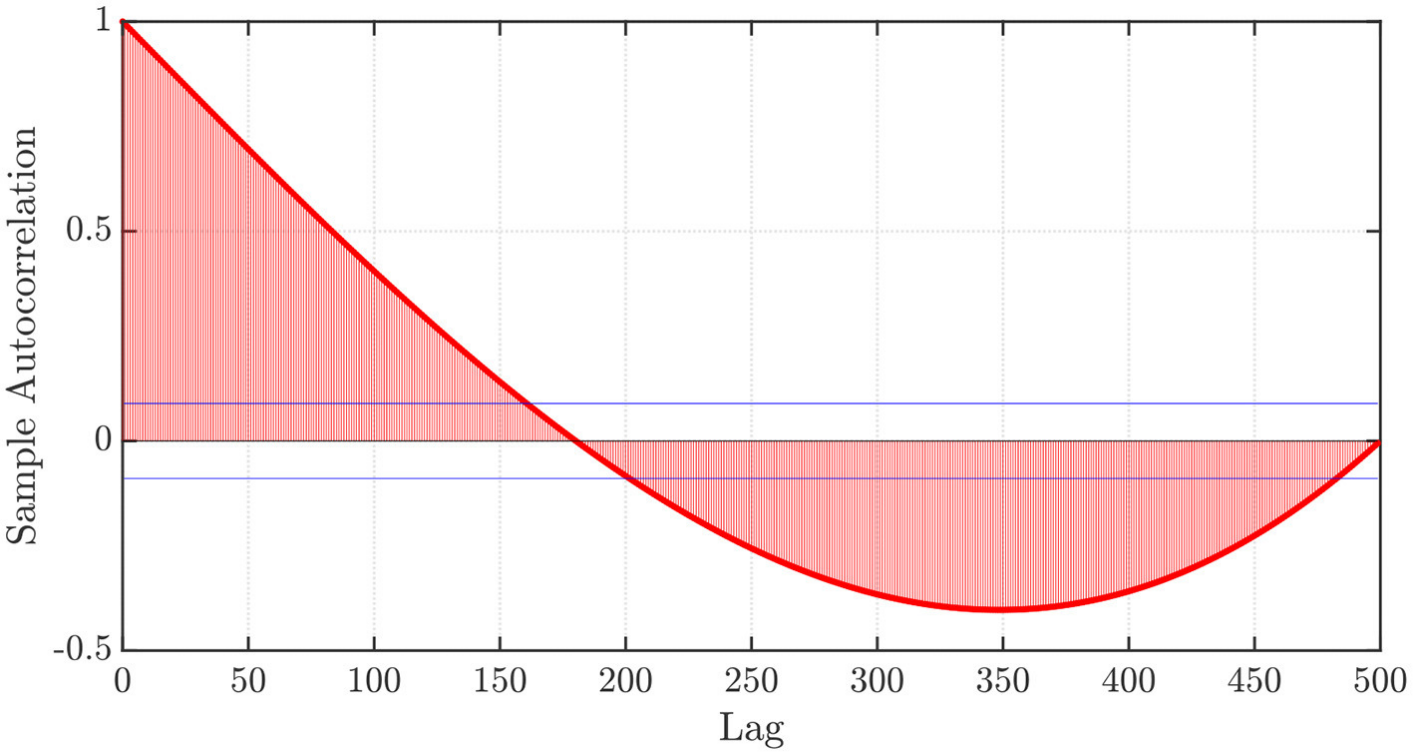
sACF plot of the functional values *f*(*x*_0_), *f*(*x*_0_ − *h*), …, *f*(*x*_0_ − (*P* − 1)*h*), with *f*(*x*) = *x*^2^, *x*_0_ = −1, *P* = 500, and *h* = 0.01.

Furthermore, let us consider an initial point *x*_0_, and let us generate a time series that considers a discretization step *h* > 0, the number of steps of memory *P* ∈ ℕ, and the corresponding functional values *f*(*x*_0_), *f*(*x*_0_ − *h*), …, *f*(*x*_0_ − (*P* − 1)*h*). Next, we investigate the sample autocorrelation function (sACF) obtained from the aforementioned values. The sACF profile is shown in Figure 1. First, we notice that the sample autocorrelation function (sACF) obtained from the aforementioned values and depicted in Figure 1 suggests slower than exponential algebraic decay and statistically significant (for a significance level of 5%) dependency on past lags, with a large area enclosed by the composite sACF curve and the horizontal axis. This suggests that the ARFIMA(*p, d, q*) processes described above can successfully predict the behavior of the functional values obtained. Furthermore, although the dependency on past lags is shown here for a quadratic function, other functions will exhibit similar behaviors, which are likely associated with properties related to the local curvature of the objective function at any given point. As such, we can use the determined (or learned) ARFIMA(*p, d, q*) model to predict the values that the function will take in a ‘descending direction’ to then consider the argument that attains the lowest predicted value. Then, we take the value of this argument as the initial point and proceed similarly to the above-mentioned point until a desirable stopping criterion is attained. It is worth mentioning that while using ARFIMA(*p, d, q*) processes, we do not explicitly need to know the function *f*(·), but instead, only the functional values.

**Figure 2:**
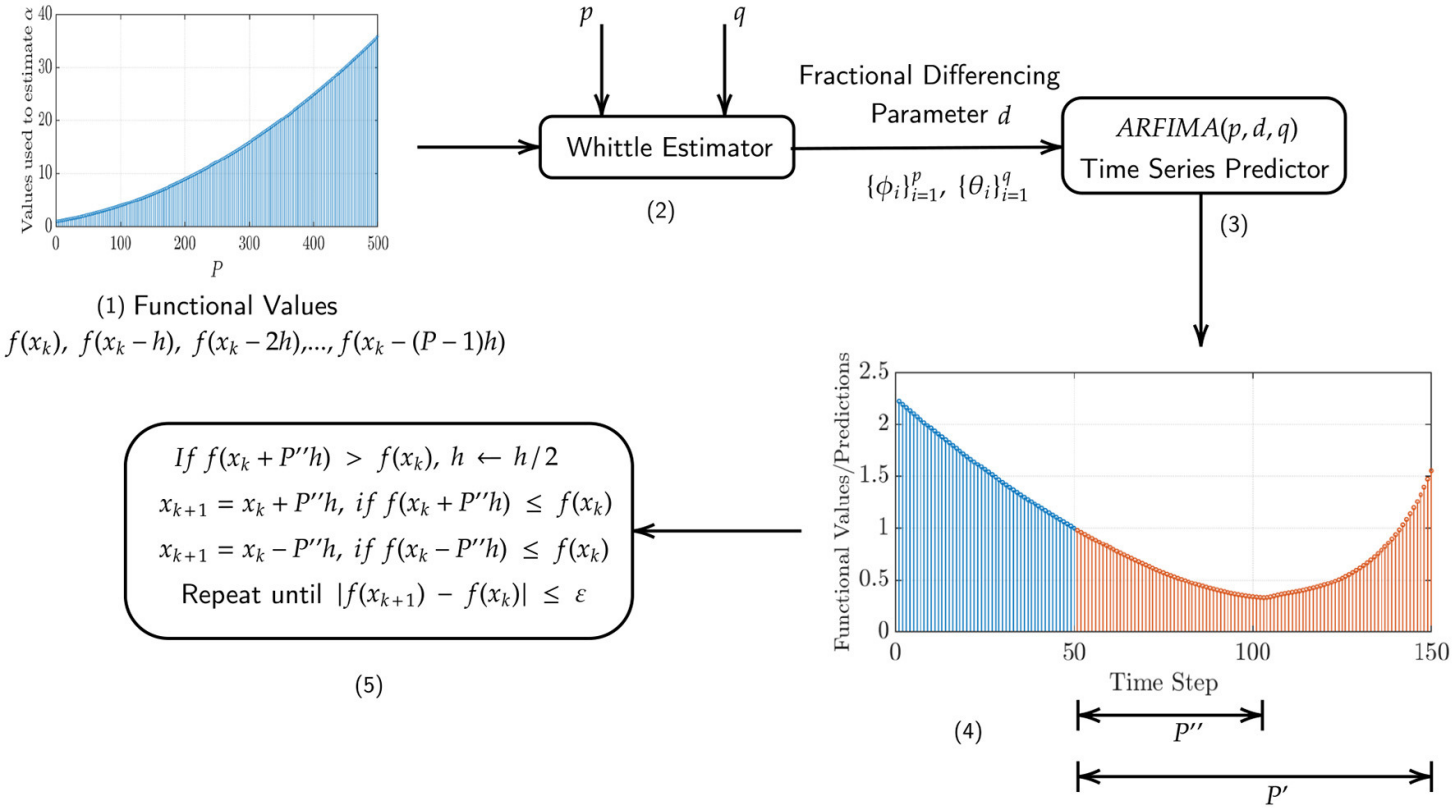
Schematic representation of the NEO method. (1) The functional values *f*(*x*_0_), *f*(*x*_0_ − *h*), …, *f*(*x*_0_ − (*P* − 1)*h*), with pre-specified values of *x*_0_, *P*, and *h*, used by the Whittle estimator. (2) The Whittle estimator, which takes the aforementioned values as input and outputs the fractional differencing parameter *d* and the autoregressive and moving average coefficients ${\left\{\phi_{i}\right\}}_{i=1}^{p}$ and ${\left\{\theta_{i}\right\}}_{i=1}^{q}$, respectively. (3) The ARFIMA time series predictor, which predicts *P*′ steps into the future. (4) Illustration of the largest possible value of *P*″ ≤ *P*′, such that *P*″ satisfies $y_{1}\geq y_{2}\geq \ldots \geq y_{P^{\prime \prime }}\leq y_{P^{\prime \prime }+1}$. (5) Update step for the iterate *x*_*k*_.

NEO is described in its general form in Algorithm 1 and a schematic overview is presented in Figure 2 for the unidimensional unconstrained case, which can be applied to solve multiple dimensional problems as well by iteratively running it over each individual dimension. In what follows, the steps corresponding to the NEO method are described in more detail.

**Algorithm 1:**
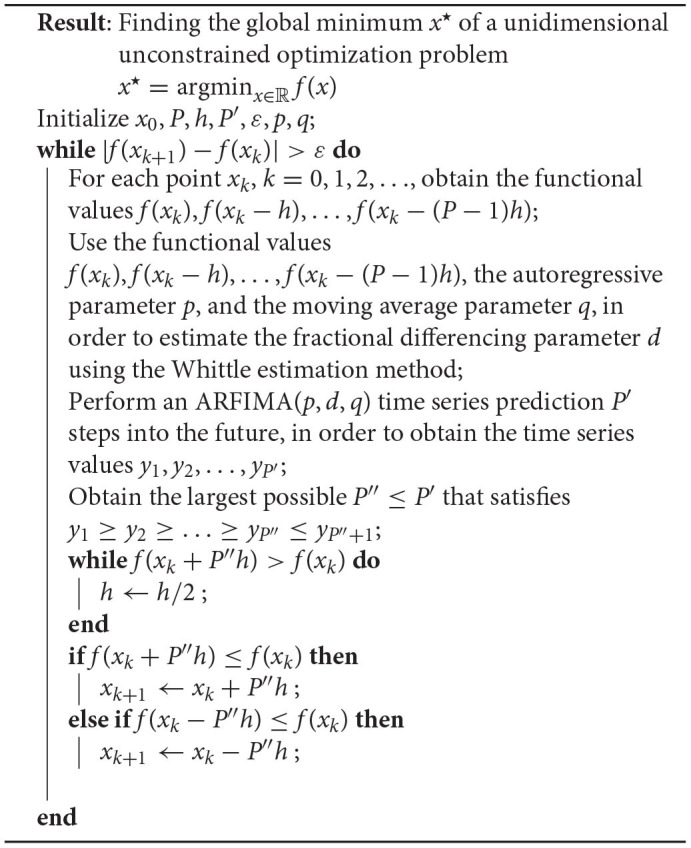
The NEO method for unidimensional unconstrained optimization problems.

First, we consider the pre-specified values of *p* and *q* along with the Whittle estimation procedure (Whittle, 1961) (see details in the Appendix in the Supplementary Material) to find the fractional differencing parameter *d* and the autoregressive and moving average coefficients ${\left\{\phi_{i}\right\}}_{i=1}^{p}$ and ${\left\{\theta_{i}\right\}}_{i=1}^{q}$, respectively, from the functional values *f*(*x*_*k*_), *f*(*x*_*k*_ − *h*), …, *f*(*x*_*k*_ − (*P* − 1)*h*), for *k* = 0, 1, 2, …. From numerical experimentation, particularly the one presented in Figure 1, we can see from the sACF of the functional values that the latter are correlated over time, which is then directly associated with the curvature of the function in the optimization landscape.

Next, we employ the above estimated parameters to perform an ARFIMA time series prediction, *P*′ steps into the future, in order to obtain the time series $y_{1},y_{2},\ldots ,y_{P^{\prime }}$. As depicted in the Figure 2, we find that our ARFIMA time series predictions are limited in their predictive capabilities since they can only capture information about the local behavior of the function upto a certain finite number of time steps into the future. Since many descent methods in the optimization literature require us to satisfy *f*(*x*_*k*+1_) ≤ *f*(*x*_*k*_), we select the largest possible value of *P*″ ≤ *P*′, such that *P*″ satisfies $y_{1}\geq y_{2}\geq \ldots \geq y_{P^{\prime \prime }}\leq y_{P^{\prime \prime }+1}$. If, at this stage, $f\left(x_{k}+P^{\prime \prime }h\right)>f\left(x_{k}\right)$, we update the discretization step *h* by *h*/2 until the condition $f\left(x_{k}+P^{\prime \prime }h\right)\leq f\left(x_{k}\right)$ is satisfied. Once that is obtained, we update the current iterate as


(8) $$x_{k+1}=\{\begin{array}{ll} x_{k}+P^{\prime \prime }h, & \text{if }f(x_{k}+P^{\prime \prime }h)\leq f(x_{k})\\ x_{k}-P^{\prime \prime }h, & \text{if }f(x_{k}-P^{\prime \prime }h)\leq f(x_{k}) \end{array}.$$

The method terminates when |*f*(*x*_*k*+1_) − *f*(*x*_*k*_)| ≤ ε, where ε ∈ ℝ^+^ is a specified tolerance.

## 4. Simulation Results

In this section, we generalize the NEO method shown for unidimensional problems in Algorithm 1 to two-dimensional problems.

### 4.1. Results on Two-Dimensional Optimization Problems

**Figure 3:**
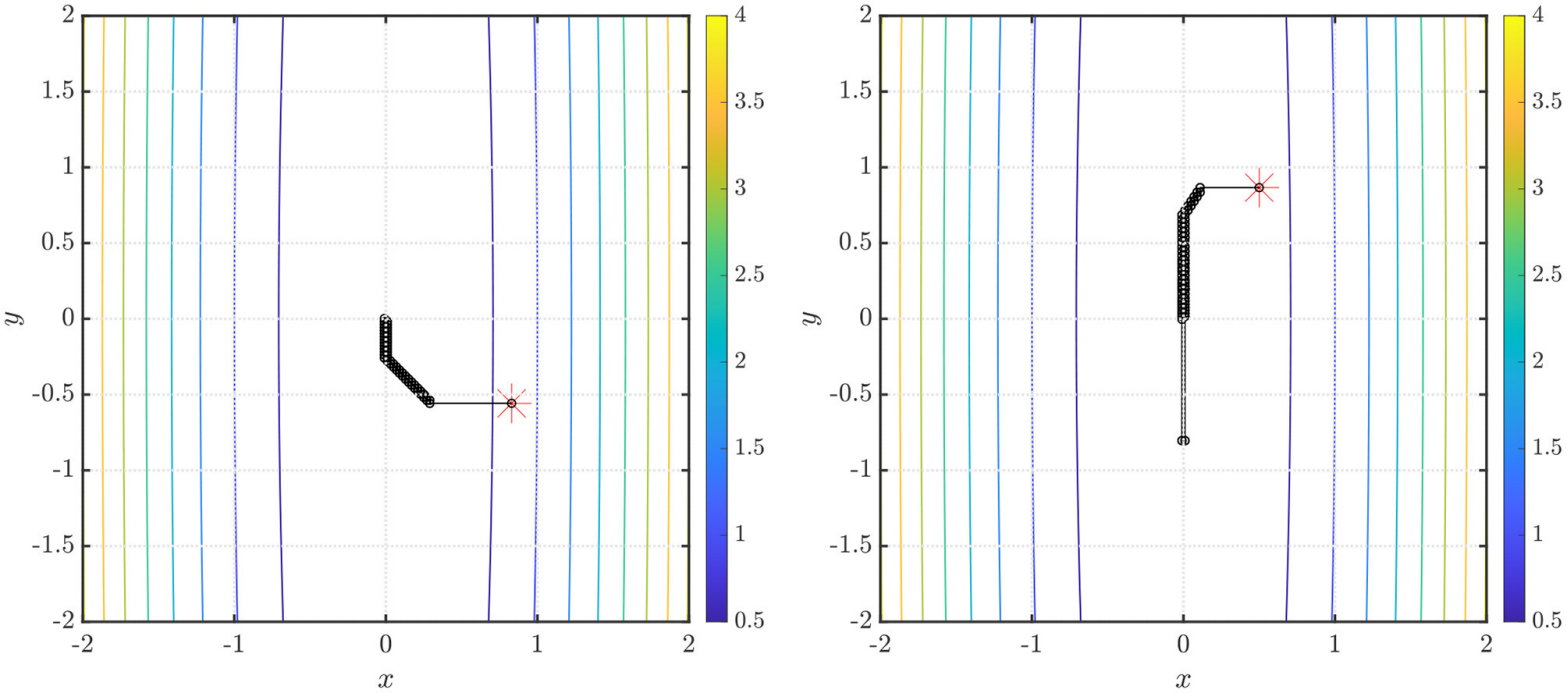
(**Left**) Steps taken in the two-dimensional plane when the NEO method is used to find the global minimum of the function *f*(*x, y*) = *x*^2^ + 0.01*y*^2^. The initial point (denoted by the red asterisk) *x*_0_ is arbitrarily selected on the unit circle. We use ARFIMA(4, *d*, 0) time series predictions. (**Right**) The initial point (denoted by the red asterisk) $x_{0}={\left[1/2\;\sqrt{3}/2\right]}^{\text{T}}$. We use ARFIMA(4, *d*, 0) time series predictions and use a constant value of *d* averaged out after the first 10 iterations of the method.

In the first example, we show the performance of our method in finding the global minimum of the function *f*(*x, y*) = *x*^2^+0.01*y*^2^ in Figure 3 (left). The starting point *x*_0_, denoted by the red asterisk, is chosen to be an arbitrary point on the unit circle. Convergence is obtained in 56 iterations using ARFIMA(4, *d*, 0) time series predictions. We use *P* = 100 steps of memory and an initial grid discretization step of *h* = 0.01, settings we will preserve for the remainder of the paper.

We also consider the problem setting where we use our method in order to find the global minimum of the functions *f*(*x, y*) = **x**^*T*^*Q***x**, with **x** = [*x* *y*]^*T*^ and *Q* = diag(1, κ), with κ ∈{1, 0.1, 0.01, 0.001}. For each case, we use 12 different equispaced starting points initialized on the unit circle. To highlight the differences, we compare our approach against gradient descent implemented with inexact backtracking line search (Bertsekas, 1997; Nocedal and Wright, 2006), which we use to tune the step size of gradient descent. The results, in terms of iterations taken to convergence as well as running time averaged over the entire run, are provided in Table 1.

**Table 1:** Iterations taken to convergence to find the minimum of *f*(*x, y*) = *x*^2^ + κ*y*^2^ with 12 different starting points *x*_0_ initialized on the unit circle, given by $x_{0}={\left[\cos\theta \sin\theta \right]}^{T}$, for values of κ in the set {1, 0.1, 0.01, 0.001}, for the NEO method vs. gradient descent (GD) implemented with inexact backtracking line search (for the same starting point in each row). ARFIMA(4, *d*, 0) time series predictions are used in our method for each case.

	**θ**	**Iterations for NEO**	**Time for NEOSys. Id. (s)**	**Time for NEO(s)**	**Iterations for GD**	**Time for GD (s)**
κ = 1	π/6	24	5.3050*e* − 01	6.6400*e* − 05	3	3.2000*e* − 03
	π/3	24	9.2580*e* − 01	5.7020*e* − 04	3	3.7000*e* − 03
	π/2	22	7.0860*e* − 01	3.0400*e* − 05	3	4.2000*e* − 03
	2π/3	9	6.3920*e* − 01	6.3700*e* − 05	30	9.6000*e* − 03
	5π/6	51	6.5400*e* − 01	7.2700*e* − 05	3	3.9000*e* − 03
	π	50	5.4640*e* − 01	2.9600*e* − 05	3	4.1000*e* − 03
	7π/6	68	5.4910*e* − 01	5.9800*e* − 05	3	5.7000*e* − 03
	4π/3	68	4.6320*e* − 01	6.4600*e* − 05	3	3.5000*e* − 03
	3π/2	50	7.2680*e* − 01	3.0700*e* − 05	3	6.1000*e* − 03
	5π/3	51	6.1120*e* − 01	8.9000*e* − 05	3	3.9000*e* − 03
	11π/6	42	5.8540*e* − 01	6.6900*e* − 05	3	4.1000*e* − 03
	2π	5	5.8140*e* − 01	2.7930*e* − 04	3	3.7000*e* − 03
κ = 0.1	π/6	39	3.5680*e* − 01	1.2690*e* − 04	24	7.4000*e* − 03
	π/3	44	5.5990*e* − 01	8.6200*e* − 05	26	7.7000*e* − 03
	π/2	21	7.1380*e* − 01	6.6500*e* − 05	25	8.9000*e* − 03
	2π/3	56	3.1770*e* − 01	6.1500*e* − 05	26	5.6000*e* − 03
	5π/6	84	8.2920*e* − 01	9.4500*e* − 05	24	6.5000*e* − 03
	π	50	4.5560*e* − 01	4.4100*e* − 05	30	6.0000*e* − 03
	7π/6	68	4.0790*e* − 01	6.3800*e* − 05	24	7.1000*e* − 03
	4π/3	68	4.9120*e* − 01	9.6300*e* − 05	26	5.7000*e* − 03
	3π/2	50	3.9610*e* − 01	4.0700*e* − 05	25	6.1000*e* − 03
	5π/3	51	3.7460*e* − 01	1.2800*e* − 04	26	6.7000*e* − 03
	11π/6	42	4.9560*e* − 01	8.6100*e* − 05	24	7.9000*e* − 03
	2π	14	4.3690*e* − 01	6.3250*e* − 04	30	6.5000*e* − 03
κ = 0.01	π/6	37	4.1270*e* − 01	6.4600*e* − 05	459	2.0000*e* − 03
	π/3	37	3.5440*e* − 01	2.4000*e* − 03	486	1.0400*e* − 02
	π/2	38	3.4640*e* − 01	3.4800*e* − 05	492	7.7000*e* − 03
	2π/3	35	2.9140*e* − 01	8.2900*e* − 05	486	1.0800*e* − 02
	5π/6	64	2.4370*e* − 01	1.1600*e* − 04	459	7.8000*e* − 03
	π	50	3.8860*e* − 01	3.1900*e* − 05	60	5.9000*e* − 03
	7π/6	64	3.5810*e* − 01	1.0760*e* − 04	459	8.000*e* − 03
	4π/3	33	4.2200*e* − 01	1.5310*e* − 04	486	6.3000*e* − 03
	3π/2	24	4.9150*e* − 01	3.0500*e* − 05	492	6.5000*e* − 03
	5π/3	40	8.0420*e* − 01	6.0420*e* − 04	486	8.7000*e* − 03
	11π/6	47	5.2720*e* − 01	6.2600*e* − 05	459	8.000*e* − 03
	2π	2	3.7580*e* − 01	7.1330*e* − 04	60	7.3000*e* − 03
κ = 0.001	π/6	41	3.4880*e* − 01	7.3300*e* − 05	3453	1.4600*e* − 02
	π/3	44	3.5140*e* − 01	9.1600*e* − 05	3728	1.8700*e* − 02
	π/2	42	5.5600*e* − 01	2.9800*e* − 05	3798	1.5800*e* − 02
	2π/3	55	3.5600*e* − 01	6.5300*e* − 05	3728	1.9800*e* − 02
	5π/6	67	4.7020*e* − 01	6.6900*e* − 05	3453	1.3100*e* − 02
	π	50	3.2680*e* − 01	1.1030*e* − 04	60	5.8000*e* − 03
	7π/6	58	7.9010*e* − 01	5.6100*e* − 05	3453	1.5100*e* − 02
	4π/3	58	2.9740*e* − 01	8.5100*e* − 05	3728	1.7800*e* − 02
	3π/2	37	3.4430*e* − 01	3.4900*e* − 05	3798	1.3600*e* − 02
	5π/3	51	4.2040*e* − 01	7.6490*e* − 04	3728	1.6800*e* − 02
	11π/6	43	3.1390*e* − 01	9.0800*e* − 04	3453	1.1100*e* − 02
	2π	5	3.6100*e* − 01	2.9000*e* − 03	60	9.2000*e* − 03

We see from Table 1 that significant advantages are gained by using our approach for more ill-conditioned optimization problems, i.e., functions with a lower value of κ. Specifically, our method performs better where small changes in the inputs to the function cause significant changes to the functional value, owing to the function being very sensitive to its inputs. From the comparative study, we also note that with our approach, we have done away with the need to have a step size as an integral component of our method, thus reducing the complexities involved in its tuning. We also do not need to use any first-order or second-order gradient or Hessian information, with only the functional values sufficing. All implementations are done in Matlab R2019b, running on an x64-based AMD Ryzen 5 2500U (with Radeon Vega Mobile Gfx) processor at 2.00 GHz, with 8 GB of RAM.

We note here that although a rich body of results is provided in this paper for two-dimensional quadratic problems, we argue that this important subclass of optimization problems actually provides a diverse setting that generalizes to more complicated scenarios. To see why this is true, consider the performance of the gradient descent method used to minimize the function *f* :ℝ^*n*^ → ℝ given by


(9) $$\begin{array}{r} f\left(x\right)=\frac{1}{2}x^{\text{T}}Ax-b^{\text{T}}x+c, \end{array}$$

where *A* ∈ ℝ^*n*×*n*^ is positive definite and *b, c* ∈ ℝ^*n*^. We assume that *LI* ≽ *A*≽ ℓ*I*, where the notation *A* ≽ *B* signifies the partial order (Loewner order) defined by the convex cone of positive semi-definite matrices and that *A* − *B* is positive semi-definite. The above problem can, of course, be analytically solved, and the unique global minimizer is *x*^⋆^ = *A*^−1^*b*. Further, *f*(*x*) is convex (actually strictly convex) since the Hessian *Hf*(*x*) = *A* ≻ 0, i.e., *Hf*(*x*) is positive definite^1^. Consider the standard gradient descent method


(10) $$\begin{array}{r} x_{k+1}=x_{k}-\eta_{k}\nabla f\left(x_{k}\right), \end{array}$$

for some starting point *x*_0_ and some sequence of step sizes {η_*k*_}. We can show that if we choose


(11) $$\eta_{k}=\frac{2}{\ell +L}\;\forall \;k,$$

then


(12) $$\begin{array}{r} ||x_{k}-x^{⋆}||\leq {\left(\frac{\kappa_{\text{GD}}-1}{\kappa_{\text{GD}}+1}\right)}^{k}||x_{0}-x^{⋆}||, \end{array}$$

where


(13) $$\begin{array}{r} \kappa_{\text{GD}}=\frac{L}{\ell }=\frac{\sigma_{\text{max}}\left(A\right)}{\sigma_{\text{min}}\left(A\right)}, \end{array}$$

where σ_max_(*A*) and σ_min_(*A*) denote, respectively, the maximum and minimum singular values of the matrix *A*. In other words, we find that even for an optimization problem in high dimensions, the maximum and minimum singular values govern the linear convergence of the constant step-size policy of gradient descent, which means that for a matrix *A* ∈ ℝ^*n*×*n*^, the convergence rate is determined by the two directions corresponding to the semiaxes of the corresponding ellipsoid in *n* dimensions.

**Figure 4:**
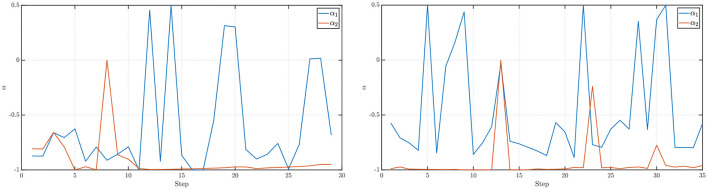
(**Left**) Values of the fractional differencing parameter α_1_ (along the *x*-axis) and α_2_ (along the *y*-axis) as estimated by the Whittle estimation procedure for the example presented in Figure 3 (left). (**Right**) Values of the fractional differencing parameter α_1_ (along the *x*-axis) and α_2_ (along the *y*-axis) as estimated by the Whittle estimation procedure for the example presented in Figure 3 (right).

We also note from the results in Table 1 that for the NEO method proposed in the paper, the identification of the fractional differencing parameter *d* (using the Whittle estimation procedure outlined in the Appendix in the Supplementary Material) seems to be the key bottleneck step when it comes to the computation of running times for the method. In practice, however, this issue can be mitigated using a scheduling approach where we use the average of the values of the parameter *d* obtained after a few time steps and then use that value instead of re-computing *d* via system identification. This approach is tried for the function *f*(*x, y*) = *x*^2^ + 0.01*y*^2^ and the results are shown in Figure 3 (right). Convergence is obtained in 70 iterations with a starting point $x_{0}={\left[1/2\;\sqrt{3}/2\right]}^{T}$ using ARFIMA(4, *d*, 0) time series predictions. Additionally, we also present, in Figure 4 (left, right), respectively, the values of the fractional differencing parameters along the *x* and *y* axis as estimated by the Whittle estimation procedure from the functional values for the case of the objective function and algorithm settings detailed in the case of Figure 3 (left, right).

### 4.2. Results on the Rosenbrock Function

In this section, we demonstrate the working of the NEO method on the Rosenbrock function (Rosenbrock, 1960), which is a non-convex function used as a test problem for a wide variety of optimization scenarios and algorithms. The non-convexity of the function as well as its global minimum lying in a narrow, flat, parabolic valley makes the minimization of the Rosenbrock function a difficult problem to solve. The function is defined by


(14) $$\begin{array}{r} f\left(x,y\right)={\left(a-x\right)}^{2}+b{\left(y-x^{2}\right)}^{2}, \end{array}$$

and has a global minimum at the point (*a, a*^2^), where *f*(*x, y*) = 0. Hereafter, we consider a commonly used set of parameters, *a* = 1 and *b* = 100 (Shang and Qiu, 2006).

**Figure 5:**
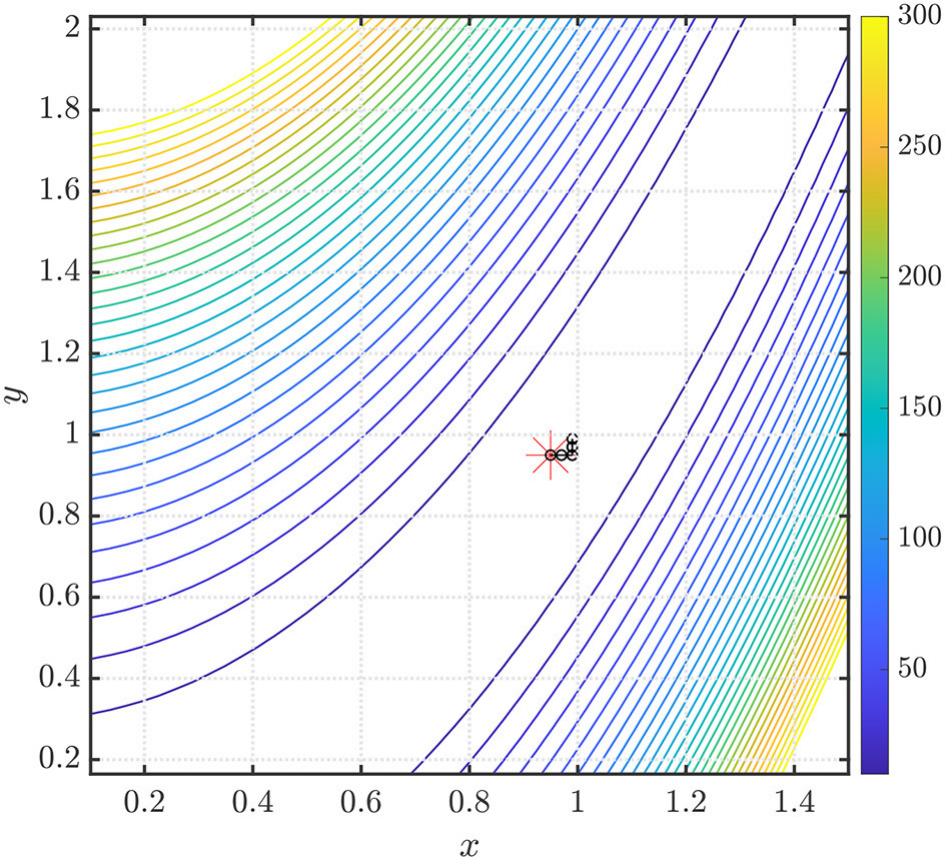
Steps taken in the two-dimensional plane when the NEO method is used to find the global minimum of the Rosenbrock function. The initial point (denoted by the red asterisk) $x_{0}={\left[0.95\;0.95\right]}^{T}$. We use ARFIMA(4, *d*, 0) time series predictions.

In Figure 5, we show the performance of the NEO method in finding the global minimum of the Rosenbrock function. The starting point *x*_0_, denoted by the red asterisk, is chosen to be the point $x_{0}={\left[0.95\;0.95\right]}^{T}$. Convergence is obtained to the point [0.99 0.97]^*T*^ in 4 iterations using ARFIMA(4, *d*, 0) time series predictions. We use *P* = 100 steps of memory and an initial grid discretization step of *h* = 0.01. It is important to note that for the Rosenbrock function, the Hessian often possesses a large condition number, which leads to the poor performance of gradient-descent-like algorithms. Additionally, any optimization method generates a unique dynamics that will be sensitive to the chosen initial point; thus, warm starts are often desirable in the context of non-convex optimization.

### 4.3. Results on Non-linear Activation Functions

In this section, we evaluate the performance of the NEO method on two non-linear activation functions. Such activation functions are found in neural networks and act as non-linearities in the same. In the same way as integrated circuits receive a multitude of signals and then make a decision regarding whether the output signal will be on or off (as a function of the input), activation functions act as a proxy for this behavior in artificial neural networks. However, when neural networks are used, the particular form of the error or loss function used (which in turn contains these non-linear activation functions), often suffers from issues of ill-conditioning (Saarinen et al., 1993; Zhang et al., 1995; van der Smagt and Hirzinger, 2012) due to function optimization landscapes that are often ridden with flat areas and saddle points.

Additionally, if methods such as Newton's method or the Levenberg-Marquardt algorithm (Levenberg, 1944; Marquardt, 1963) are used to minimize the loss function, there is additional dependence on the conditioning of the Jacobian or the Hessian matrix evaluated at each of the iterates, and hence, there is considerable loss of performance if either of the Jacobian or the Hessian matrices are ill-conditioned or rank-deficient. In what follows we look into a simple yet meaningful example where non-linear activation functions found in neural networks are minimized using the NEO method.

Assume that we have the activation function


(15) $$\begin{array}{r} \varrho_{\text{GELU}}\left(s\right)=\frac{s}{2}\left(1+\mathrm{erf}\left(\frac{s}{\sqrt{2}}\right)\right), \end{array}$$

where the *error function* $\mathrm{erf}z=\frac{2}{\sqrt{\pi }}\int_{0}^{z}e^{-t^{2}}\text{ d}t$ for any *z* ∈ ℂ. This activation function is known in the literature as a Gaussian Error Linear Unit (GELU) (Hendrycks and Gimpel, 2016). Accordingly, for a set of weights *w*_1_ and *w*_2_ and for a set of inputs *x*_1_ and *x*_2_, we assume that the GELU activation function acts on the weighted sum *w*_1_*x*_1_ + *w*_2_*x*_2_ in order to produce


(16) $$\begin{array}{r} \varrho_{\text{GELU}}\left(w_{1}x_{1}+w_{2}x_{2}\right)=\frac{w_{1}x_{1}+w_{2}x_{2}}{2}\left(1+\mathrm{erf}\left(\frac{w_{1}x_{1}+w_{2}x_{2}}{\sqrt{2}}\right)\right). \end{array}$$

We use the NEO method in order to minimize (16) and find the weights *w*_1_ and *w*_2_ when *x*_1_ = *x*_2_ = 1. Using *P* = 100 steps of memory, an initial grid discretization step of *h* = 0.01, ε = 10^−3^, with *P*′ = 100 steps ahead ARFIMA(4, *d*, 0) time series predictions, we obtain convergence in 23 iterations with the optimal values *w*^⋆^ = [−0.5142 − 0.2188]^*T*^ and $\varrho_{\text{GELU}}^{⋆}\;=\;-0.1699$.

**Figure 6:**
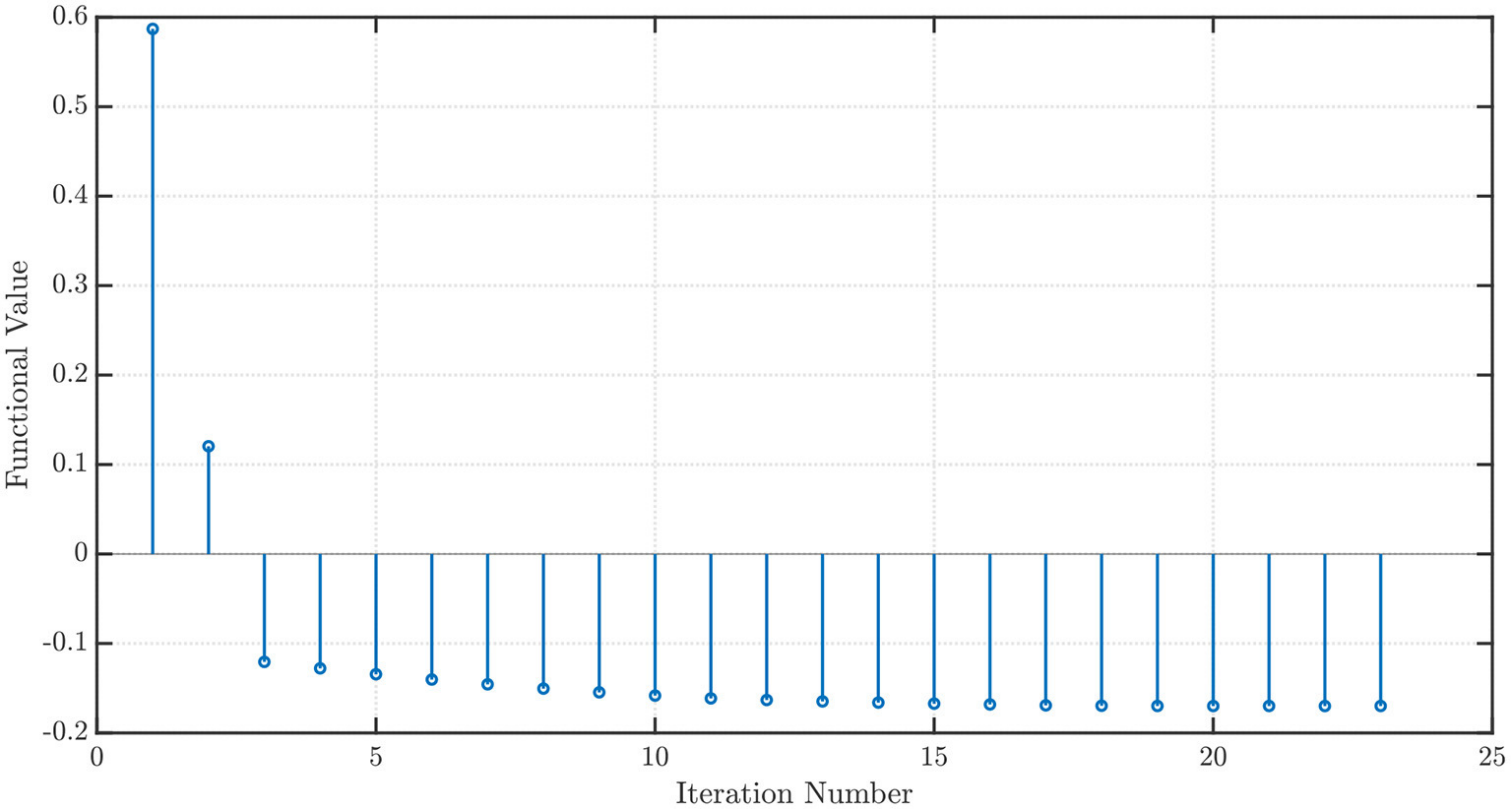
Convergence profile of the NEO method minimizing the Gaussian Error Linear Unit (GELU) activation function.

**Figure 7:**
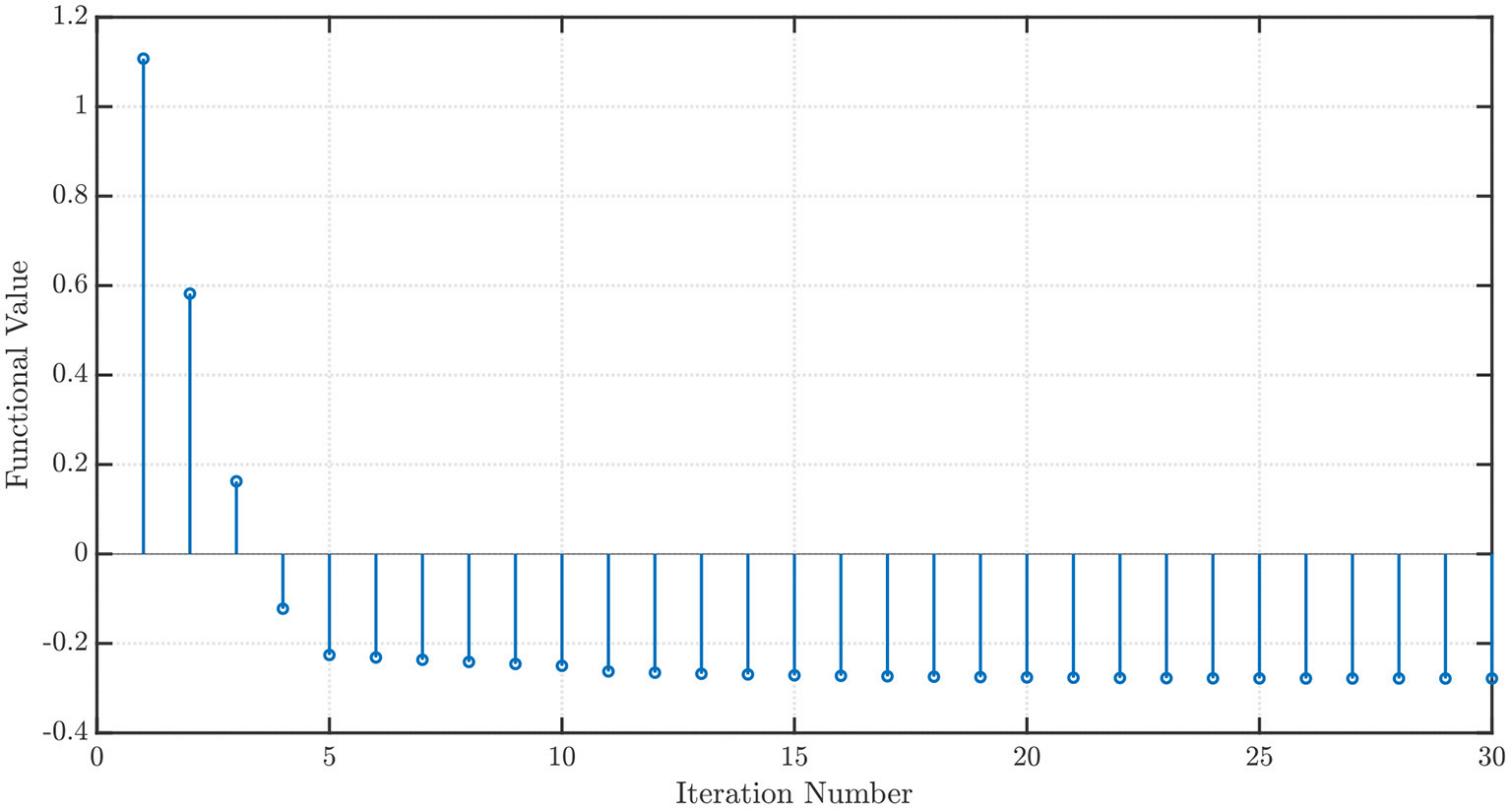
Convergence profile of the NEO method minimizing the Sigmoid Linear Unit (SiLU) activation function.

Additionally, we also use the NEO method to minimize the activation function


(17) $$\begin{array}{r} \varrho_{\text{SiLU}}\left(s\right)=\frac{s}{1+e^{-s}}, \end{array}$$

with


(18) $$\begin{array}{r} \varrho_{\text{SiLU}}\left(w_{1}x_{1}+w_{2}x_{2}\right)=\frac{w_{1}x_{1}+w_{2}x_{2}}{1+e^{-\left(w_{1}x_{1}+w_{2}x_{2}\right)}}, \end{array}$$

which is called the Sigmoid Linear Unit (SiLU) (Elfwing et al., 2018) or Swish-1 (Ramachandran et al., 2017). Once again, with *x*_1_ = *x*_2_ = 1, *P* = 100 steps of memory, an initial grid discretization step of *h* = 0.01, ε = 10^−3^, with *P*′ = 100 steps ahead ARFIMA(4, *d*, 0) time series predictions, we obtain convergence in 30 iterations with the optimal values *w*^⋆^ = [−2.1316 0.8362]^*T*^ and $\varrho_{\text{SiLU}}^{⋆}=-0.2784$. The convergence profiles when the NEO method is used to minimize the GELU and the SiLU activation functions are shown in Figures 6, 7, respectively.

## 5. Discussion and Conclusions

In this paper, we introduced a NEuro-inspired Optimization (NEO) method, that is motivated by the neurophysiological modeling capabilities possessed by fractional calculus-based ARFIMA time series models to determine an approximation of the argument that minimizes a given unconstrained optimization problem. Our proposed method does not require the computation of gradient or Hessian information, or explicitly tuning a step size, an issue that, in spite of receiving widespread coverage in the optimization literature, still proves to be a bottleneck in the design of such algorithms.

Future work will entail the automated selection of the autoregressive and moving average orders from the functional values at every time step [based on the Akaike Information Criterion (AIC) (Akaike, 1974) or the Bayesian Information Criterion (BIC) (Schwarz, 1978)] and the automation of determining the fractional-order coefficient, which proved to be the most computationally intensive step in our approach, in a wider range of optimization benchmarks.

It is also interesting to note here the connections between descent-like methods such as NEO with proximal algorithms that are used to solve non-differentiable optimization problems (Parikh and Boyd, 2014). The latter aims to consider a regularizer with an additional quadratic term to be optimized, which adds smoothness to the optimization problem to be explored. That being said, it is similar to NEO, which seeks to explore memory and local changes in the functional values to smooth the predictions. Additionally, it can be shown that under some mild technical assumptions, one can use averaged proximal operators and algorithms in order to convert minimization problems into fixed-point iterations, much like NEO looks at minimization problems from the iterative step descent point-of-view. It would, therefore, be interesting to establish the relationship between proximal algorithms and the NEO method based on the aforementioned relationships. Lastly, we believe that the validation of our approach in the context of a variety of real-world applications would also be interesting to look into.

## Data Availability Statement

The original contributions generated for the study are included in the article/supplementary material, further inquiries can be directed to the corresponding author/s.

## Author Contributions

SC, SD, and SP performed the research. SC was responsible for the execution of the numerical experiments and wrote the manuscript with revisions by SD and SP. All authors contributed to the article and approved the submitted version.

## Funding

The authors gratefully acknowledge the support by the National Science Foundation under Grant Number CMMI 1936578.

## Conflict of Interest

The authors declare that the research was conducted in the absence of any commercial or financial relationships that could be construed as a potential conflict of interest.

## Publisher's Note

All claims expressed in this article are solely those of the authors and do not necessarily represent those of their affiliated organizations, or those of the publisher, the editors and the reviewers. Any product that may be evaluated in this article, or claim that may be made by its manufacturer, is not guaranteed or endorsed by the publisher.

## Footnotes

^1^An *n* × *n* symmetric real matrix *A* is said to be positive definite (respectively, positive semi-definite) if *x*^*T*^*Ax* > 0 (respectively, *x*^*T*^*Ax* ≥ 0) for all non-zero *x* in ℝ^*n*^.
