# Supplementary material for: NEO: NEuro-Inspired Optimization—A Fractional Time Series Approach
Source: Front Physiol. 2021 Sep 21;12:724044. doi: 10.3389/fphys.2021.724044 (PMC8491743; doi:10.3389/fphys.2021.724044)
Supplement: Supplementary file 1 [file Data_Sheet_1.pdf]

Supplementary Material

APPENDIX

ON THE SKETCH OF A CONVERGENCE PROOF FOR THE NEO METHOD

At each stage of the NEO method, we perform a time series prediction with an ARFIMA(p, d, q) model P' steps into the future, the predicted values being given by $y_1, y_2, \dots, y_{P'}$. In a descent framework, we need to satisfy the condition $f(x_{k+1}) \le f(x_k)$ at each step of the optimization algorithm. For the NEO method, we select the largest possible $P''_k \le P'$ such that we have $y_1 \ge y_2 \ge \dots \ge y_{P''_k} \le y_{P''_k+1}$. The NEO method (for each dimension) then boils down to the following update step along dimension i

$$x_{k+1} = x_k \pm P''_k h \mathbf{e}_{i,k}, \tag{S1}$$

where $\mathbf{e}_{i,k}$ for $1 \le i \le n$ (n being the dimension of the objective function $f : \mathbb{R}^n \to \mathbb{R}$) represents the standard basis vector with a 1 in coordinate i and 0's elsewhere, and the choice of the positive or negative sign in (S1) is made according to whether $f(x_k + P''_k h) \le f(x_k)$ or $f(x_k - P''_k h) \le f(x_k)$, respectively. We can visualize (S1) as a generalized descent step with the rate of descent given by $P''_k h$ and the direction of descent at iteration k given by the standard basis vector $\mathbf{e}_{i,k}$. If $f(x)$ is continuously differentiable, i.e., $f(x) \in C^1$, convergence of the NEO method follows under mild technical assumptions holding for the step size $P''_k h$ and the descent direction $\mathbf{e}_{i,k}$ by leveraging the results in Lemma 3.1 and Theorem 3.1 of Wu (1992).

WHITTLE ESTIMATION OF THE FRACTIONAL EXPONENT

The maximum likelihood estimation of the fractional differencing parameter d for an ARFIMA process was first introduced by Whittle (Whittle, 1961; Fox and Taqqu, 1986; Choudhuri et al., 2004). Since then, frequency-domain approaches to estimating the fractional differencing parameter d have been preferred over other approaches since they are invariant to unknown means of the ARFIMA time series under consideration (Cheung and Diebold, 1994).

We start with a sample of T observations of a stationary, uniformly spaced, centered process $y_1, \dots, y_T$ and a sequence of angular frequencies defined by

$$\omega_j = \frac{2\pi j}{T} \tag{S2}$$

for $j = 1, 2, \dots, m$. For $m \ll T$, the Discrete Fourier Transform (DFT) of the sequence y_t is given by

$$\widehat{c}(\omega_j) = \frac{1}{\sqrt{2\pi T}} \sum_{k=1}^{T} y_k e^{i\omega_j k}. \tag{S3}$$

The Whittle estimator utilizes the fact that the coefficients $\widehat{c}(\omega_j)$ are asymptotically independent Gaussian random variables with mean value zero and variance given by the spectral density of the process at that frequency (Baum et al., 2020). We first define the quantities

$$I(\omega_j) = |c(\omega_j)|^2 \tag{S4}$$

and

$$f_y(\omega_j) = \frac{1}{2\pi} \sum_{k=-\infty}^{\infty} \gamma_k e^{-i\omega_j k}, \tag{S5}$$

which are, respectively, the sample periodogram and the spectral density at ω_j, with γ_k being the autocovariance at lag k. With the definitions in (S4) and (S5), the likelihood function for the estimation procedure is given by

$$L_j = \frac{1}{\sqrt{2\pi f_y(\omega_j)}} \exp\left\{ -\frac{I(\omega_j)}{2 f_y(\omega_j)} \right\}. \tag{S6}$$

It can be shown that at low frequencies, the spectral density $f_y(\omega)$ satisfies

$$\lim_{\omega \to 0^+} \omega^{2d} f_y(\omega) = G, \tag{S7}$$

where $G = G(d) > 0$. From the above expression, we see that the time series process y_t has finite power provided $2d < 1$, and, hence, d provides us with a measure of the long-term memory of the process y_t. Replacing $f_y(\omega_j)$ with its asymptotic approximation $G\omega_j^{-2d}$ from (S7), we get the following expression for the negative log-likelihood

$$-\log L_j(G, d) = \frac{1}{2} \left\{ \log 2\pi + \log G - 2d \log \omega_j + \frac{1}{G} \omega_j^{2d} \left| \widehat{c}(\omega_j) \right|^2 \right\}. \tag{S8}$$

This expression is used in the Whittle estimation of the fractional differencing parameter d. Alternatively, we can also adopt a local approach (Kunsch, 1987; Robinson et al., 1995), where the m lowest angular frequencies $\omega_1, \ldots, \omega_m$ are considered, and the Whittle estimate for d is obtained by minimizing the negative log-likelihood function

$$-\log L(G, d) = \frac{m}{2} \left\{ \log 2\pi + \log G - \frac{2d}{m} \sum_{j=1}^{m} \log \omega_j + \frac{1}{G} \frac{1}{m} \sum_{j=1}^{m} \omega^{2d} \left| \widehat{c}(\omega_j) \right|^2 \right\}. \tag{S9}$$

Taking partial derivatives of (S9) with respect to G gives us the optimal value of G for any given d as

$$\widehat{G}(d) = \frac{1}{m} \sum_{j=1}^{m} \omega_j^{2d} \left| \widehat{c}(\omega_j) \right|^2, \tag{S10}$$

with the local Whittle estimator minimizing the function

$$R(d) = \log \widehat{G}(d) - \frac{2d}{m} \sum_{j=1}^{m} \omega_j, \tag{S11}$$

with $\widehat{G}(d)$ being defined by the expression

$$\widehat{G}(d) = \frac{1}{m} \sum_{j=1}^{m} \omega_j^{2d} I(\omega_j). \tag{S12}$$

REFERENCES

Baum, C. F., Hurn, S., and Lindsay, K. (2020). Local Whittle estimation of the long-memory parameter. *The Stata Journal* 20, 565–583

Cheung, Y.-W. and Diebold, F. X. (1994). On maximum likelihood estimation of the differencing parameter of fractionally-integrated noise with unknown mean. *Journal of Econometrics* 62, 301–316

Choudhuri, N., Ghosal, S., and Roy, A. (2004). Contiguity of the Whittle measure for a Gaussian time series. *Biometrika* 91, 211–218

Fox, R. and Taqqu, M. S. (1986). Large-sample properties of parameter estimates for strongly dependent stationary Gaussian time series. *The Annals of Statistics* , 517–532

Kunsch, H. R. (1987). Statistical aspects of self-similar processes. In *Proceedings of the First World Congress of the Bernoulli Society, 1987* (VNU Science Press), vol. 1, 67–74

Robinson, P. M. et al. (1995). Gaussian semiparametric estimation of long range dependence. *The Annals of Statistics* 23, 1630–1661

Whittle, P. (1961). Gaussian estimation in stationary time series. *Bulletin of the International Statistical Institute* 39, 105–129

Wu, S. (1992). Convergence properties of descent methods for unconstrained minimization. *Optimization* 26, 229–237
